# Microwave Spectroscopic Detection of Human Hsp70 Protein on Annealed Gold Nanostructures on ITO Glass Strips

**DOI:** 10.3390/bios8040118

**Published:** 2018-11-27

**Authors:** Rodica Elena Ionescu, Raphael Selon, Nicolas Pocholle, Lan Zhou, Anna Rumyantseva, Eric Bourillot, Eric Lesniewska

**Affiliations:** 1 Light, Nanomaterials and Nanotechnology (L2N), FRE-CNRS 2019, Institute Charles Delaunay (ICD), University of Technology of Troyes, 12 Rue Marie Curie CS 42060, 10004 Troyes CEDEX, France; lan.zhou@utt.fr (L.Z.); anna.rumyantseva@utt.fr (A.R.); 2 Laboratory Interdisciplinaire Carnot de Bourgogne (ICB), UMR-CNRS 6303, University of Bourgogne Franche-Comté, 9 Avenue Alain Savary, 21078 Dijon CEDEX, France; raphael.selon@u-bourgogne.fr (R.S.); nicolas.pocholle@u-bourgogne.fr (N.P.); Eric.Bourillot@u-bourgogne.fr (E.B.); lesniew@u-bourgogne.fr (E.L.)

**Keywords:** biosensor, ITO functionalization, label-free microwave detection, molecular chaperone Hsp70

## Abstract

Conductive indium-tin oxide (ITO) and non-conductive glass substrates were successfully modified with embedded gold nanoparticles (AuNPs) formed by controlled thermal annealing at 550 °C for 8 h in a preselected oven. The authors characterized the formation of AuNPs using two microscopic techniques: scanning electron microscopy (SEM) and atomic force microscopy (AFM). The analytical performances of the nanostructured-glasses were compared regarding biosensing of Hsp70, an ATP-driven molecular chaperone. In this work, the human heat-shock protein (Hsp70), was chosen as a model biomarker of body stress disorders for microwave spectroscopic investigations. It was found that microwave screening at 4 GHz allowed for the first time the detection of 12 ng/µL/cm^2^ of Hsp70.

## 1. Introduction

Scanning microwave microscopy (SMM) [1] combines the principles of atomic force microscopy (AFM) with microwave frequency measurements of conductive or non-conductive materials. The typical frequency range of microwave spectroscopy is chosen between 1–18 GHz [2]. This type of microscopy belongs to near field probing techniques.

Among the different fields of application, such as 3D tomography [3] detection of light chemical element profile [4], SMM remains a powerful tool for surface characterization in nanotechnology. However, no real use of the microwave spectroscopy to confirm the specific (bio)functionalization of solid metallic active supports is reported. Thus, the authors focus their current work on the potential use of the microwave spectroscopy technique to characterize the modification of annealed gold–(ITO) glass substrates with (bio)molecules for the detection of human 70 kDa heat-shock proteins (Hsp70). Heat shock proteins [5] are an important part of the cellular machinery responsible for protein folding and protecting the cell from stress. In particular, the Hsp70 protein is either overexpressed on the surface of cancer cells or in the peripheral blood (for example, in diabetic patients), constituting a central component of the cellular network of molecular chaperones [6,7], and plays a role in numerous biological events, including protein folding and assembly, post-translational modification, transportation, secretion, inflammation [8], and degradation.

Recent advances in non-destructive nanoscale surface quantum research, based on high speed atomic force microscopy (HS-AFM) coupled with surface enhanced Raman spectroscopy (SERS), enable high-speed imaging of molecular chaperones such as Hsp Lo18 or Hsp70 and their chemical recognition, which is useful for the development of biosensors (unpublished data). The HS-AFM instrument allows imaging at a rate of 5–20 frames/s (fps) without disturbing the functioning of fragile proteins and sensitive protein–protein and DNA–protein interactions. The field of exploitation of this prototype of high-speed AFM in biology is vast, because the additional dimension of the temporal evolution of biological processes is evaluated (structural and functional dynamics). The authors focused on the study of membrane-related processes (important in signaling, transport, and energy generation), protein–DNA complexes [9] (important in gene regulation and transcription process), and the effect of the surface nature and confinement on the selectivity, reactivity, and biological activity of proteins [10] (interactions between proteins and inorganic materials) important for the development of the biosensor.

**Figure 1:**
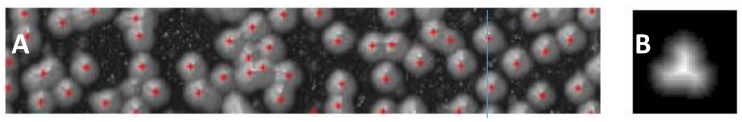
High speed atomic force microscopy (HS-AFM) image of heat shock proteins (Hsp70) deposited on indium-tin oxide (ITO) substrate. (**A**) Automation of information extraction from HS-AFM images with extraction of center for density calculation (size 250 × 45 nm). (**B**) Calculated shape form extracted from 250 protein analysis.

Beyond the imaging of supramolecular assemblies, there remains a question to be answered: What is the surface effect on the thermodynamic properties of bio-molecules? The goal was achieved by the nanoscopic understanding of ionic selectivity, activity, chemical reactivity, and conformational modifications of bio-molecules *in vitro,* depending on the geometry of the confined support and of its physico-chemical properties. Flat and charged surfaces have been selected to bind proteins. Protein–substrate interactions can be adjusted by altering the type and concentration of ions in solution, by varying the temperature, and chemical modifications of the surface. Flat and nanostructured surfaces (with and without proteins) could be characterized with HS-AFM in solution [11]. From the information on the image, we can obtain information on the geometry (Figure 1): transitions, local variations, connectivity, topology, morphology, presence of known shape models, and statistical and frequency characterization.

The influence of pH and the concentration of proteins in inducing a conformational change has also been studied (data not shown). From these studies, we decided to characterize the ITO biochip dedicated to the detection of tumor biomarkers using five different concentrations. Full coverage is obtained for the median concentration.

## 2. Materials and Methods

### 2.1. Chemicals and Biomolecules

The 70 kDa heat shock proteins in 20 mM Tris-HCl pH 7.5 and 2 mM DTT at a concentration of 1 mg/mL were provided by Prospec company (ref. HSP-170, Rehovot, Israel). HSP70 kDa produced in *Escherichia coli* is a single, non-glycosylated polypeptide chain (1–641 amino acids—a.a.) containing 661 a.a. fused to a 20 a.a. His-tag at the N-terminus and having a total molecular weight (MW) of 72.2 kDa. Hsp70 was stored at 4 °C if the entire vial will be used within 2–4 weeks. Before each experiment, the Hsp70 proteins were suspended in different volumes of Tris(hydroxymethyl)aminomethane hydrochloride buffer (Tris-HCl), pH 7.5, purchased from Sigma-Aldrich company. 11-Mercaptoundecanoic acid (MUA) and *N*-hydroxysuccinimide (NHS) were provided by Sigma-Aldrich.

### 2.2. Instruments for the Characterization of Gold Nanoparticles on Glass Strips

**Figure 2:**
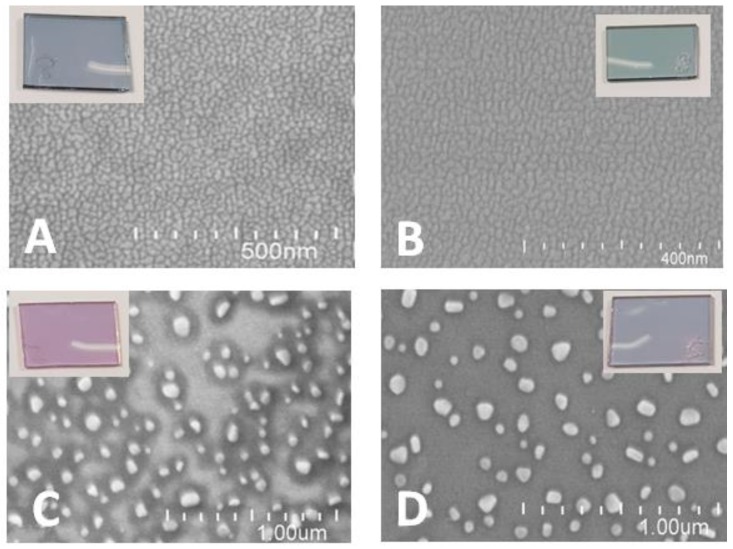
SEM images of gold films (4 nm) as evaporated (**A**) on glass substrate; (**B**) on ITO substrate and after annealing at 550 °C, for 8 h (**C**) on glass substrate (**D**) or ITO substrate. Insets-photos of real glass samples.

SEM: The morphology of gold films deposited on non-conductive and ITO-conductive substrates and after thermal annealing was systematically analyzed by scanning electron microscopy (FEG-SU8030, Hitachi, Tokyo, Japan). For example, Figure 2A–D shows the morphology of the evaporated and annealed specific gold layers (4 nm) on different substrates, respectively.

**Figure 3:**
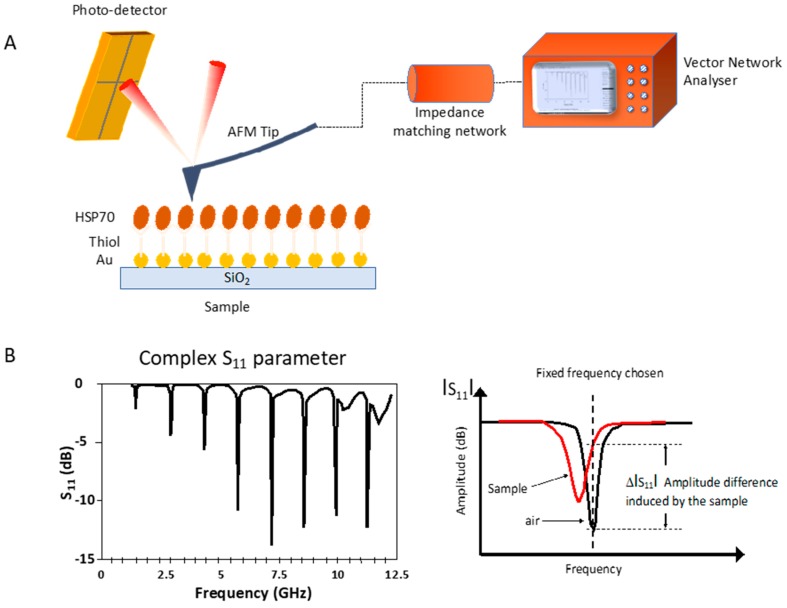
Experimental set-up for microwave spectra experiments. AFM tip-to-sample distance control and measured amplitude signal (**A**). S_11_-the complex reflection coefficient (**B**).

SMM: The analyses of the samples were performed by scanning microwave microscopy (AFM 5600 LS, Santa Rosa, CA, USA) from Keysight Technologies. The SMM typically operates at microwave frequencies from 1 MHz to 18 GHz. The SMM set-up is shown in Figure 3A. Built on the AFM FESP high-performance probe with a nominal spring constant of 3 N/m, Bruker’s SCM-PIT probe features an electrically conductive Platinum-Iridium-coated tip (Pt-Ir), ideal for electrical characterization applications. The Pt-Ir coating on the front side of the cantilever provides a metallic electrical path from the cantilever die to the apex of the tip. The cantilever and the probe act as a local radiant antenna for emission and reception of the microwave signal.

Microwave spectroscopy consists of measuring the amplitude of microwave signal reflection for each frequency of the spectrum, allowing the physical properties to be detected in the test sample. A 2-port Vector Network Analyser 1 MHz–18 GHz(VNA N5230A, Keysight Technologies, Santa Rosa, CA, USA) associated to a specific impedance matching resonator circuit measures the ratio of the incident and reflected signal at the tip, the so-called scattering S_11_ parameter (Figure 3B).

Measurement using microwave microscopy can detect the local changes of various materials’ properties, such as conductivity or chemical composition. In the present case, elements of various chemical compositions were tested by varying their concentration. These different chemical compositions induce a modification of the dielectric constant which will be quantified with respect to the response in the air. This differentiation will be done by choosing the signal in the air as the fixed reference frequency, which will be compared to the signal in the presence of the sample in contact with the tip. The amplitude variation of this difference ΔS_11_ will give the impact of the sample on the microwave signal (Figure 3B). In order to increase the spatial detection to be compatible with nano-sized objects, the device uses a near-field technique thanks to an AFM tip close to the sample. The S_11_ parameter depends on the dielectric permittivity value of the medium in interaction with the microwaves. As a consequence, all the modifications such as bio-functionalization, molecule absorption, molecule grafting, DNA hybridization, and immunoreactions, which all affect the permittivity of the medium, will be easily detected. The detection principle is based on the comparison, at a given frequency, between the S_11_ value in air and the S_11_ value in presence of the sample being tested.

### 2.3. Preparation of Gold Nanostructures on Non-Conductive and Conductive Substrates

Large-scale annealed gold nanoparticles were fabricated on glass in four steps: cleaning, drying on hot plate at 100 °C, gold evaporation, and oven annealing at 550 °C. Thus, soda lime (SiO_2_) non-conductive and ITO conductive substrates were used for gold film deposition [12]. Before metal evaporation, all glass substrates were cleaned with a mixed solution of detergent (Decon90) and deionized water (ddH_2_O) in an ultrasonic water bath at 50 °C for 15 min. Further, the glass samples were thoroughly rinsed with ddH_2_O, dried under N_2_ steam, subjected to ultrasonication in deionized water at 50 °C for 5 min three times, and then rinsed with fresh deionized water. Finally, the substrates were dried again under a N_2_ steam and transferred on a hotplate at 100 °C for 10 min, followed by exposure to metal evaporation in a vacuum evaporator (Plassys MEB400, FR, Marolles-en-Hurepoix, France). For one set of gold evaporation, five glass strips were placed together on a support-plate inside the evaporation chamber under a lower pressure of 1 × 10^−6^ Torr. The metal deposition rate was maintained at 0.01 nm/s by changing the value of the working current during the rotation of the support with the glass samples to obtain a homogeneous deposition of gold films on the non-conductive and conductive substrates. For microwave spectroscopy studies, the influence of three gold thicknesses (2 nm, 4 nm, and 8 nm) on functionalization and detection of Hsp70 protein were investigated.

Finally, the gold (Au)-modified glasses were transferred to a high-temperature oven (Model N.60/65A; No. 171,693; T_max_ = 650 °C, Nabertherm, Germany) and annealed at a high temperature of 550 °C for 8 h. The Au samples were characterized by SEM (Figure 2).

### 2.4. Biofunctionalization of Gold Nanostructures on Glass and ITO Substrate

The annealed gold nanostructures on glass substrates were exposed to 150 µL MUA-thiol (8 mM) dissolved in ethanol for 1 h at 4 °C in a petri dish used as a humid chamber. After 1 h, the samples were rinsed with ethanol, dried, and incubated with 100 µL NHS-solution (4.5 mg/mL) for 20 min, then rinsed with the Tris-buffer. Moreover, five activated gold NPs substrates were biofunctionalized for each protein concentration over 30 min with 2 µL of human heat-shock proteins (hHsp70) solutions of a given concentration at room temperature and successively rinsed with Tris-buffer and ddH_2_O. Five concentrations of human heat-shock proteins (55, 40, 25, 20, and 12 ng/µL/cm^2^, respectively) were detected with the microwave spectroscopy at room temperature (23.5 °C) with a relative humidity (RH) of 32%.

### 2.5. Microwave AFM Measurements

**Figure 4:**
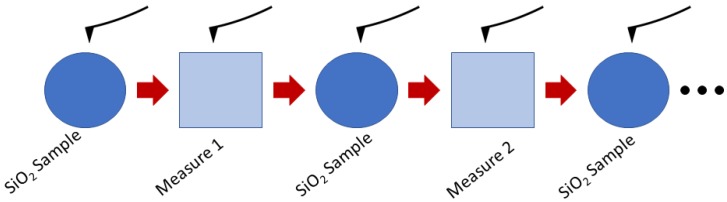
Step-by-step microwave AFM measurement protocol.

The microwave measurement protocol established to monitor the impact of each element of the nanochips development is illustrated in Figure 4. Microwave spectrum acquisition is obtained by point-to-point recording of data from each area of interest. Each sample analysis is preceded and followed by an analysis of the SiO_2_ reference substrate. Having a homogeneous structure, in thickness and component, allows us to check that no drift appeared during the measurement phase on the samples or to take it into account if necessary. Thus, to ensure the reproducibility of the measurements, nine measuring points are made on each specific area as described in Figure 4. Twenty-resonance frequency spectra are implemented to determine which one will obtain the best sensitivity and stability.

## 3. Results and Discussion

### 3.1. Size Distribution of Gold Nanoparticles on Different Substrates

**Figure 5:**
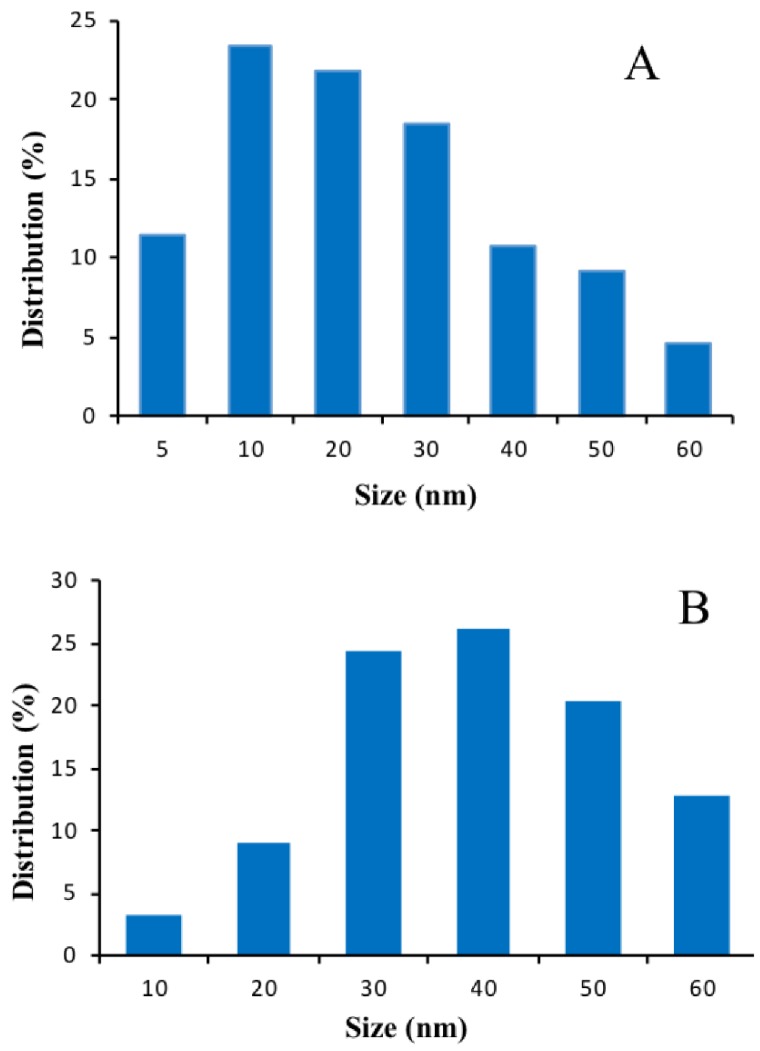
Annealed AuNPs (4 nm) on glass substrate (**A**) and on ITO substrate (**B**).

The size distribution of annealed AuNPs on non-conductive and conductive substrates are shown in Figure 2C,D and analyzed using the Public Domain ImageJ software developed by the National Institutes of Health. The results are described in Figure 5A,B.

It should be noted that on glass substrates, a wide AuNPs size distribution has been observed in a range of 5 to 30 nm, with a majority of NPs of 10 nm to 30 nm range (Figure 5A), while the size distribution of AuNPs deposited on ITO substrate is narrow in a range from 30 to 40 nm (Figure 5B). It shows that size distribution of 10 nm AuNPs (23.4%) on glass substrate is greater than that of AuNPs on ITO substrate (3.2%). Moreover, the percentage of size distribution on glass substrate is 23.4% (10 nm) > 21.8% (20 nm) > 18.4% (30 nm) > 11.5% (5 nm). However, the percentage of size distribution of 40 nm AuNPs (26.2%) on ITO glass is the highest compared to that of other size distributions, and the percentage of the second has a size distribution of 30 nm (24.3%).

### 3.2. Microwave Spectroscopy Measurements for 2 nm, 4 nm, and 8 nm Au–Glass Samples

As previously introduced in the microwave measurement protocol, nine spectral acquisitions are performed on each component, respectively SiO_2_ alone, SiO_2_ plus gold nanoparticles, SiO_2_/AuNPs/thiols, and finally SiO_2_/AuNPs/thiols and different protein concentrations (Figure 3). Twenty spectral acquisitions are arbitrarily selected from nine different areas on the surface of each sample and averaged. In order to choose the appropriate frequency for the SMM measurements, a frequency sweep in the range of 1 GHz to 10 GHz was performed with 64,001 acquisition points. The quantity to be evaluated, amplitude (dB), is the value ΔS_11_ which is plotted as a function of the frequency. All microwave measurements are performed under a controlled atmosphere (22 °C, 35% RH).

**Figure 6:**
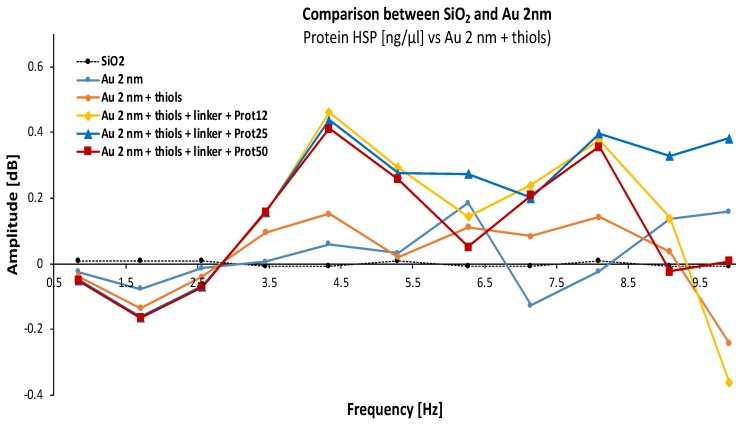
Spectral characterization of materials with gold film thickness of 2 nm for different Hsp70 protein concentrations (12, 25, and 50 ng/μL/cm^2^, respectively).

In this first case (Figure 6), we compare the amplitude signal ΔS_11_ as a function of the frequency for each functionalization step of the biochip with gold film thickness of 2 nm, for different concentrations of Hsp70 proteins (12, 25, and 50 ng/μL/cm^2^). Starting from the SiO_2_ signal, it can be observed that among the large number of resonances visible in the graph, the addition of each chemical entity changes the microwave signal. Indeed, each building modifies the equivalent impedance of the sample and therefore the dielectric constant of the studied material at the frequencies used. However, for each frequency, there is not necessarily a linear consistency in the sensing response depending on the different molecules added. Only the frequency of 4.33 GHz seems to give a “linear” response according to the different elements for this studied system.

**Figure 7:**
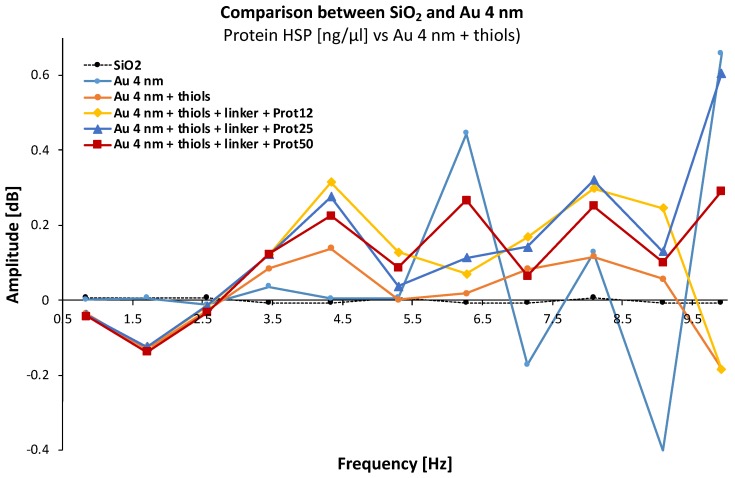
Spectral characterization of materials with gold film thickness of 4 nm for different Hsp70 protein concentrations (12, 25, and 50 ng/μL/cm^2^).

We have reissued the same measurement protocol with a gold film thickness of 4 nm (Figure 7). Again, the microwave signal varies depending on the addition of each chemical entity. It is also found that only the frequency of 4.33 GHz seems to give a “linear” response according to the different elements of the studied system.

**Figure 8:**
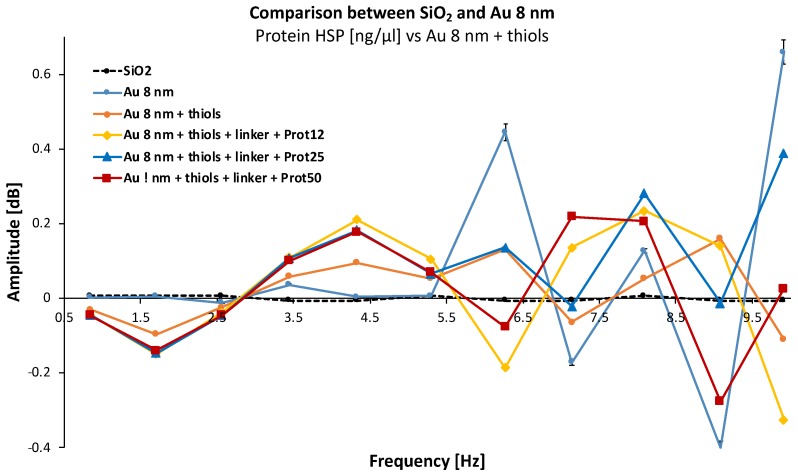
Spectral characterization of materials with gold film thickness of 4 nm for different Hsp70 protein concentrations (12, 25, and 50 ng/μL/cm^2^).

Finally, SMM measurements on gold film thickness of 8 nm (Figure 8) were also performed. The conclusion is identical to the two previous cases but with a slightly difference that we will discuss.

### 3.3. Optimization of Microwave Measurements Using Three Independent Gold Thicknesses on Glass Samples

**Figure 9:**
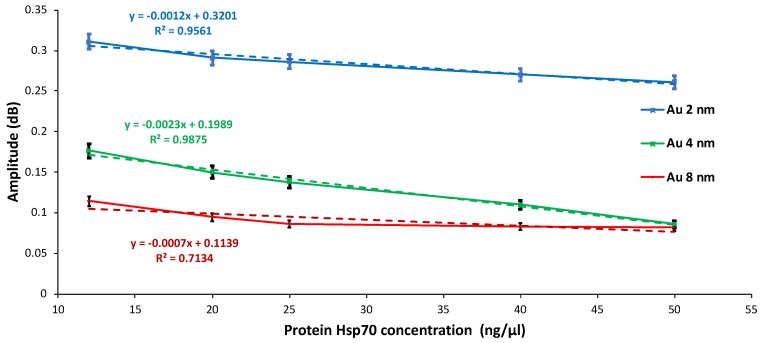
Calibration curves of Hsp70 protein deposited on gold nanostructures (2 nm, 4 nm, and 8 nm) at 4.33 GHz.

Among the different tested frequencies (range from 1 GHz to 10 GHz) for gold films of three thicknesses (2 nm, 4 nm, and 8 nm) evaporated on glass, the optimal microwave (bio)sensing results were obtained at 4.33 GHz (Figure 9). Five concentrations of Hsp70 were tested in this work. For a given concentration, except the highest one (50 ng/µL/cm^2^ for Au 4 nm and Au 8 nm), the amplitude of the signal decreases as the size of nanoparticles increases.

In addition, it is important to note that the 2 nm and 4 nm Au-modified substrates show a linear evolution of amplitude (dB) (R^2^ = 0.96 for Au 2 nm and 0.99 for Au 4 nm) for five protein concentrations Hsp70 tested (ng/µL/cm^2^) with a slope value equal to −13.10^−4^ and to −23.10^−4^ dB/(ng/µL) for Au 2 nm and Au 4 nm.

It is worth mentioning that the slope of the curve is higher for 4 nm gold-modified glass, indicating that the sensibility is better for this particle size. For samples of Au 8 nm, a saturation amplitude signal was obtained for protein concentrations greater than 25 ng/µL/cm^2^. It was also found that the amplitude measurements had a linear evolution for a lower protein concentration in the range of 50 ng/µL/cm^2^.

## 4. Conclusions

For the first time, detection of changes in the concentration of Hsp70 protein was performed using microwave spectroscopy. Glass substrates modified with annealed gold nanoparticles were prepared and studied according to the different thicknesses of evaporated metal and the sizes obtained between the neighboring nanoparticles after annealing. Thus, the SMM technique has clearly demonstrated its capacity and sensitivity to follow each step of the (bio)functionalization of the biochip (SiO_2_/AuNP, SiO_2_/AuNP/Thiols, and SiO_2_/AuNP/Thiols/Hsp70) and statistically confirmed the uniformity of the SMM responses. In particular, for a frequency of 4 GHz, the SMM technique allowed us to follow the linear evolution of the variation of the protein concentration with a sensitivity of 12 ng/μL/cm^2^.
